# Chronic Intestinal Auto-Intoxication*Read before the International Medical Society of Mexico, Section in General Medicine, January 24, 1908. Chairman’s Address.

**Published:** 1908-03

**Authors:** R. H. L. Bibb

**Affiliations:** Saltillo


For the Texas Medical Journal.

### Chronic Intestinal Auto-Intoxication.*

BY R. H. L. BIBB, M. D., SALTILLO.

Since Bouchard's classical publications, in 1887 and in 1890, intestinal auto-intoxication has challeged attention from the foremost medical men and investigators of the age, and it is regarded, today, as one of the most potent factors in the causation of many of the diseases which physicians are called upon to treat. In view of the admitted importance of the subject, then, it could hardly be expected that, in an address for this occasion, the author would be able to do more than pass, in rapid review, the most important features of the subject, did ability permit him to deal with it otherwise.

Chronic intestinal auto-infection has been traced, almost exclusively, to faulty changes in the albumins and nucleins within the alimentary canal, due to some defect in pancreatic digestion, aided by bacterial influences, wherein albumoses are formed from the albumins, and uric acid is formed from the nucleins—bacterial action being spent, principally, if not wholly, upon the albumins, so that "pancreatic putridity," instead of the products of pancre-

*Read before the International Medical Society of Mexico, Section in General Medicine, January 24, 1908. Chairman's Address.

atic digestion, are carried into the colon where they are converted, by the fauna and the flora of that portion of the digestive tube, into indol and phenol, and other less noxious compounds, which enter the system through the blood vessels and lacteals, and which are, for a time, that is variable, as this period is determined by the surroundings and other conditions which tend for or against the health and well-being of the individual, filtered out, oxidized, or rendered inert by the liver, the lymphatics, the kidneys, the the lungs, muscles, brain, skin and other organs of the body until some one or more of these organs break down and the victim becomes a patient under the care of his medical adviser, too often, long after irreparable damage has been done.

A greater or lesser amount of putridity is thought to be associated with the digestion of even those regarded as of good health; but, as a rule, it may be stated that the amount of indol and phenol, xanthin, hypoxanthin, paraxanthin, etc., will be found in a given case, will depend upon the nature of the food supply, its preparation, quantity, mastication, digestion, etc.; the time it remains in the colon—for it is this portion of the digestive tract that is richest in bacteria—as well as upon the number and nature of its contained flora.

Radiating from the intestines to every cell throughout the human economy, as might be expected, the symptoms of *chronic intestinal auto-intoxication* are multiform, and, oftentimes, exceedingly perplexing. Thus, of 77 cases analyzed by Forchheimer—37 males and 40 females—the complaint was general in 12 cases; 18 complained of gastric troubles; 12 of intestinal derangement; 52 had disorder of the nervous system of some sort; 4 disease of the genito-urinary organs; 4 had skin affections; 17 had locomotor difficulties; 7 had defects of the eyes, nose or ears. Sixty-six of the 77 had Riggs' disease (pyorrhea alveolaris); 8 had healthy gums, and 3 were toothless. On closer analysis, Forchheimer found that 73 of 77 patients presented evidence of bowel derangement; 27 were dyspeptics; 19 had hyperchlorhydria; 1 achlorhydria; 4 sensory or motor gastric neuroses; 1 had gastroptosis; 18 were regular in their evacuations; 4 were irregular; 44 were constipated; 5 had diarrhea; 5 diarrhea alternating with constipation; 3 suffered from flatulence and colic; 5 had enteritis mucosa, and 10 had chronic appendicitis.

In a large proportion of cases, basing this assertion on personal observation, there is greater or lesser derangement in the function of the kidneys. In some cases there is marked polyuria, so that the physician may suspect glycosuria, diabetes insipidus or fibroid kidney; in others, oliguria, is equally pronounced, while yet in other cases the two symptoms may alternate. The specific gravity of the urine may vary from 1009 to 1030, and indican, in excess, is present in a very large majority of all cases. In 68 of Forchheimer's 77 cases, it was increased; and in 39 of his 44 constipated cases indican was not only increased, but in all of his cases—13—where the colon was filled with fecal matter, the greatest indican index obtained. The quantity of indican was also augmented in all of Forchheimer's cases where constipation alternated with diarrhea; and, contrary to what might have been expected, in view of the supposed poisonous action of that substance on the renal cells, in only 6, out of his 77 cases, did Forchheimer find indican associated with albumin in the renal secretion. In 600 urinary examination made by Williams—who is disposed to question that indican, itself, is prejudicial to the individual, but who admits its importance as indicating intestinal putrefaction, found it associated with albumen in 103 cases, and present in 108 cases where albumen was absent, or 211 times in the 600 examinations. Luff believes that kidney disease and gout both owe their origin to the influence of toxins which are, in all probability, produced in, and absorbed from, the intestinal tract. "It can not be denied," he writes, "that the classic remedies for gout have only two things in common: one, that they relieve gout; and, the other, that they check intestinal putrefaction, or diminish the absorption of its products or promote their elimination from the system." He continues: "The intestinal tract is a very powerful factor, if not the primary factor in the development of gout," and he builds up a plan of medicinal, dietetic, balenological and climatic management of the disease, based upon this idea.

Nervous symptoms were manifest in 61 of Forchheimer's 77 cases; 6 of whom had genuine migraine; 11 had neuralgia; 2 suffered from neuritis; 4 from paresthesia; 14 were nervous and irritable; 7 were nervous, irritable and excitable; 7 were depressed; 3 were phobiacs; 11 had insomnia; 2 were hypochondriacs; 1 was melancholic; 4 were hysterical, and 4 had vertigo. In 58 of these cases there were abnormalities in the cardio-vascular apparatus; of which 17 were neurotic; 11 had myocardial affections; 6 chronic myocarditis; 3 dilated hearts; 1 the heart of arteriosclerosis; 1 obese heart; 12 had valvular lesions; 4 aortic insufficiency; 5 mitral insufficiency; 1 mitral insufficiency and stenosis; 1 aortic and mitral insufficiency and stenosis; 3 soft systolic aortic bruit, and in 5 there was accentuation of the aortic second sound.

In analyzing the symptoms of those of the 77 cases who had affections of the locomotor apparatus, Forchheimer found that 50 had diseases of the muscles and joints, of which 19 had gouty joints; 24 had muscular rheumatism; 13 complained of transitory rheumatic muscular pains; 15 of the 19 with gross manifestations of gout had increased indican output, as did also 20 of the 24 who had muscular rheumatism. The average quantity of indican being 3.5, and the maximum, in one case, was 5 per cent. Eleven out of the 13 who had transitory muscular pains had indicanuria; 22 presented skin manifestations; 5 urticaria; 5 eczema; 3 furunculosis; 4 acne vulgaris; 1 angioneurotic edema; 1 dermatomyositis; 1 erythemata, and 1 had red axillary perspiration. Thirteen of the 77 had respiratory involvement, of which 5 had tuberculosis; 2 bronchitis; 4 bronchitis and asthma, and 2 had emphysema.

In the main, my observations agree with those of Forchheimer's, and I have elected to use the latter, for this occasion, because they are more numerous and more extensively analyzed than the former, and because of their more authoritative origin. I will take occasion to state, however, that in 30 undoubted cases of chronic intestinal auto-intoxication observed and analyzed by myself, the urine of 8 contained albumen; pyorrhea alveolaris, more or less extensive, gastro-intestinal involvement and indicanuria were present in all of them—the indican output being greatest in the most constipated; nervous phenomena, insomnia, neuralgias, and migraine was marked in 6, of which 2 had epileptiform convulsions and 1 severe and persistent vertigo; 7 were rheumatics—muscular and articular; 3 had skin eruptions, 1 urticaria and 2 acne vulgaris; 1 was asthmatic, and 5 complained of general illness, rather, general ill-health, with slight variations in the evening and morning temperatures. These 5 had been variously diagnosed, and had been treated for chronic malaria, influenza, tuberculosis, typhomalarial and typhoid fever and chronic hepatitis. Two of these cases, notwithstanding physical and microscopical examinations were constantly negative, and the indican output was as constantly high, could be diagnosed only after I had made several radio-scopic examinations of the chest and found that both sides lighted up normally, and that the incursions and excursions of the diaphragm were equal; it being about three-quarters of an inch on both sides.

From the foregoing it would appear that the diagnosis of chronic intestinal auto-intoxication is beset with many difficulties, but these difficulties are more seeming than real; for, when it can not be made by direct examination, it can almost invariably be made by exclusion. It must be remembered that indican is generally present in the urine, in increased quantity, in Addison's disease, in cholera, carcinoma of the liver, central and peripheral disease of the nervous system, typhoid fever, dysentery, carcinoma of the stomach, ileus, acute, subacute and chronic gastritis, acute and chronic peritonitis, empyema, putrid bronchitis, gangrene of the lungs, or, in short, in any condition (von Jaksch) where albuminous putrefaction is progressing, actively, in some part of the system.

From Forchheimer's observations, and from my own, intestinal disorders, Riggs' disease and indicanuria, are the three leading symptoms of chronic intestinal auto-intoxication, and I believe that a diagnosis based upon these three symptoms would be well-founded and correct.

In the treatment of chronic intestinal auto-infection, it is absolutely necessary to put the mouth and stomach in a good condition; to withdraw, at least temporarily, all albuminous food; to clean out and disinfect the alimentary tract; to prescribe gastric and intestinal tonics, and to insist upon proper food, in suitable quantities, at regular hours, that has been properly prepared and thoroughly masticated. The use of wines, coffee, tea, condiments and tobacco, and the use of fluids, in excess, during meals, should be interdicted. Oftentimes a change of diet and habits will be all that many cases will require, provided their mouths are in condition to properly masticate their food.

A pill containing two grains and a half each of blue mass and calomel, to be followed in ten or twelve hours by an ounce of castor oil, will be beneficial, at the beginning of treatment in most cases, whatever may be the condition of the stomach and bowels. As an aid to digestion, a capsule containing one-fourth grain of extract of nux vomica, and two and a half grains each of diastase and pancreatine, immediately after each meal, will benefit many. Constipated cases will require aloes, strychnine and belladonna, associated with either the galvanic, faradic, or high-frequency current. Where there is gastroptosis, motor insufficiency, or colitis mucosa, gastric and intestinal lavage with either static electricity, or the faradic or high-frequency current, will be of signal service. Salol is a safe and an efficient intestinal antiseptic, when given after meals, and, where there is diarrhea, will usually be all that is required to correct that condition in the majority of cases needing it.

---

RHEUMATIC HYPERTROPHIED HEART.—Thompson advises rest in bed; aconite; firm strapping of left side for dyspnea due to chest adhesions; belladonna with compound spirit of ether for dyspnea of mitral stenosis.—*Denver Med. Times.*

